# Investigation of Protein and Epitope Characteristics of Oats and Its Implications for Celiac Disease

**DOI:** 10.3389/fnut.2021.702352

**Published:** 2021-09-29

**Authors:** Gyöngyvér Gell, Zsuzsanna Bugyi, Christakis George Florides, Zsófia Birinyi, Dalma Réder, Zsuzsanna Szegő, Edina Mucsi, Eszter Schall, Katalin Ács, Bernadett Langó, Szandra Purgel, Katalin Simon, Balázs Varga, Gyula Vida, Ottó Veisz, Sándor Tömösközi, Ferenc Békés

**Affiliations:** ^1^Department of Biological Resources, Agricultural Institute, Centre for Agricultural Research, EötvösLoránd Research Network, Martonvásár, Hungary; ^2^Department of Applied Biotechnology and Food Science, Research Group of Cereal Science and Food Quality, Budapest University of Technology and Economics, Budapest, Hungary; ^3^Murdoch University, College of Science, Health, Engineering and Education, Perth, WA, Australia; ^4^Cereal Research Non-Profit Ltd., Szeged, Hungary; ^5^First Pest Mill and Bakery Ltd., Budapest, Hungary; ^6^Cereal Breeding Department, Agricultural Institute, Centre for Agricultural Research, EötvösLoránd Research Network, Martonvásár, Hungary; ^7^FBFD PTY Ltd., Sydney, NSW, Australia

**Keywords:** avenin, ELISA, HPLC, epitope prediction, celiac disease, oat

## Abstract

The use of pure oats (oats cultivated with special care to avoid gluten contamination from wheat, rye, and barley) in the gluten-free diet (GFD) represents important nutritional benefits for the celiac consumer. However, emerging evidence suggests that some oat cultivars may contain wheat gliadin analog polypeptides. Consequently, it is necessary to screen oats in terms of protein and epitope composition to be able to select safe varieties for gluten-free applications. The overall aim of our study is to investigate the variability of oat protein composition directly related to health-related and techno-functional properties. Elements of an oat sample population representing 162 cultivated varieties from 20 countries and the protein composition of resulting samples have been characterized. Size distribution of the total protein extracts has been analyzed by size exclusion-high performance liquid chromatography (SE-HPLC) while the 70% ethanol-extracted proteins were analyzed by RP-HPLC. Protein extracts separated into three main groups of fractions on the SE-HPLC column: polymeric proteins, avenins (both containing three subgroups based on their size), and soluble proteins, representing respectively 68.79–86.60, 8.86–27.72, and 2.89–11.85% of the total protein content. The ratio of polymeric to monomeric proteins varied between 1.37 and 3.73. Seventy-six reversed phase-HPLC-separated peaks have been differentiated from the ethanol extractable proteins of the entire population. Their distribution among the cultivars varied significantly, 6–23 peaks per cultivar. The number of appearances of peaks also showed large variation: one peak has been found in 107 samples, while 15 peaks have been identified, which appeared in less than five cultivars. An estimation method for ranking the avenin-epitope content of the samples has been developed by using MS spectrometric data of collected RP-HPLC peaks and bioinformatics methods. Using ELISA methodology with the R5 antibody, a high number of the investigated samples were found to be contaminated with wheat, barley, or rye.

## Introduction

Celiac disease (CD) is an autoimmune disorder triggered by the consumption of gluten proteins of, primarily, wheat, rye, and barley in a part of the population with certain genetic predispositions. The pathological processes induced by gluten in these individuals cause villous atrophy in the small intestines. The disease manifests in a range of symptoms from nutrient malabsorption to reproduction problems. The prevalence of CD is, on average, 1% worldwide, making it one of the most common food-related adverse reactions. Currently, the only way to treat CD is to adhere to a lifelong gluten-free diet **(**GFD) (1, 2). By omitting staple cereals, a GFD represents a risk of decreased intake of vitamins (predominantly, B group vitamins), important minerals (zinc, magnesium, selenium, and iron), and dietary fiber. The GFD is, generally, also accompanied by an excess intake of proteins, fats and sugars. Thus, the GFD must always be constructed with the help of a trained healthcare professional to aim for nutritional balance (3, 4).

Consumption of oats carries a number of nutritional benefits, including high contents of bioactive compounds such as β*-*glucans and antioxidants along with vitamin E and avenanthramides, as well as being an important source of proteins, fats, vitamins, minerals, fibers, phenolic acids, flavonoids, sterols, and phytic acid (5–8). Several clinical studies confirm that the soluble fiber β*-*glucan is strongly related to lowering blood cholesterol (LDL) levels (9–11). It can stimulate the immune system as well and positively affects the functioning of the human intestinal flora. Since oats are one of the best sources of fatty acids among the cereals, especially linoleic acid and low amounts of saturated fat, it plays a great role in reducing the risk of cardiovascular diseases (12, 13). The Food and Drug Administration of the United States of America has allowed a health claim for an association between consumption of diet, which is high in oatmeal, oat bran, or oat flour and has reduced the risk of coronary heart disease (14). This opened the era of novel utilization of oats in human nutrition as a key component in gluten-free diet (GFD) (15, 16) and as oat protein isolates, a cheap and valuable protein source for the food industry (17).

The benefits of both applications of oats as human food sources are directly related to the protein composition of the oats used, producing these food products: the inclusion of oats in the diet of celiac patients has been a controversial issue. Oats are a less likely candidate to trigger CD due to their protein composition. On the other hand, all of the important techno-functional properties of oats are directly related to the ratio of polymeric and monomeric proteins in the sample.

Wheat prolamins are the key players in the formulation of CD, especially their α- and γ-gliadin subunits (18, 19). These proteins contain a number of T cell stimulatory epitopes, mostly in their repetitive regions (20–22). In the case of oats, the main storage proteins are the 11S- and 12S-type globulins that consist approximately 80% of the total protein content. The remaining fractions are water-soluble albumins (14–20%) and the alcohol-soluble prolamins, named avenins (4–14%), depending on the genotype (23).

Oats are, in general, considered to have low CD-triggering potential due to their lower prolamin content, higher digestibility, and lower affinity to MHC (Major Histocompatibility Complex) molecules associated with CD compared with that of wheat prolamins (24).

A range of clinical studies has taken place to investigate the safety of oats in the GFD. Despite inconsistent results, a growing body of evidence concludes that the consumption of oats in moderate amounts (20–25 g/day for children and 50–100 g/day for adults) is safe for most patients with celiac in remission (25–29). A major problem of oat consumption in the celiac context is that gluten contamination from other gluten-containing cereals occurs frequently during conventional agricultural and food-processing practices (30, 31). The problem is being addressed in several countries by developing agricultural and industrial procedures to produce oats free from gluten contamination, referred to as pure oats (32–35). In line with the findings described above, the inclusion of pure oats in the GFD in moderate amounts is recommended by multiple countries, including the EU (36), the U.S. (37), and Canada (38). The legal gluten-free threshold of 20 mg/kg gluten applies to these oat products as well.

Although pure oats are considered to be safe for most patients with celiac, there are a number of studies suggesting that oats may be able to trigger CD on their own, but only affected the minority of the population with celiacs connected to individual sensitivity and the condition of the intestine (39). In a study by Lundin et al. (40), conducting a 12-week oat challenge, 18 out of 19 patients tolerated oats well. However, a single patient developed complete villous atrophy. This patient produced T cells that showed affinity to avenins and were used to identify two avenin epitopes (PYPEQEEPF and PYPEQEQPF) that may have been responsible for triggering villous atrophy. These results were limited to this single patient, but they raised questions about the presence of celiac-related epitopes in oat avenins.

According to the results of Silano et al. (41), laboratory and clinical tests with a large number of patients and a control group proved that differences can occur based on certain oat genotypes and individual sensitivity of patients as well. In the tests, duodenum segments derived from patient and control subjects were examined by fluorescent microscopy after incubation with protein extracts from different oat genotypes. Increased gliadin-induced transglutaminase enzyme production was observed on the segments incubated with protein extracts of wheat and certain oat genotypes. This suggests that not only the contamination of oats with other gluten-containing grains can cause problems, but there are oat cultivars that contain protein sequences that are low risk for patients with celiac. Based on the study of Real et al. (42), there is a great variety of potential immune reactivity of oat cultivars, which can generate a higher or lower degree of immune response in patients with celiac disease.

The contradictory preclinical and clinical results and the findings of research aimed at the genetic variability of avenin immunoreactivity (41, 43) suggest that oat varieties are not created equal in terms of their safety in CD. It has important implications for pure oat production and highlights the importance of screening oat cultivars for the presence of celiac-related avenin epitopes. Fric et al. (27) found that the monoclonal antibody G12 developed for gluten detection (44, 45) cross-reacts with some sequences in avenins, but these peptides were considered irrelevant regarding the presence or absence of the clinically proven toxic internationally agreed celiac epitopes. The researchers suggested it may be a suitable tool for a fast, high-throughput prescreening of oat varieties (46). However, the G12 do not recognize the internationally confirmed oat avenin epitopes (47), but the antibody response is well correlated with the results of T cell proliferation and interferon γ release (46). The results of the clinical studies did not support the *in vitro* measures; the reasons could be that avenins did not contain any proteolytically resistant peptides longer than 10 amino acids, and avenin peptides have low-binding stability on HLA-DQ2.5 (48).

However, to obtain reliable information about the presence of celiac-related epitopes, immunological results should be accompanied by data on protein composition. The current scientific status about the safety of oats does not provide arguments to categorize certain oat cultivars as really harmful regarding CD. LC-MS (liquid chromatography–mass spectrometry) is the most important tool for the identification and quantification of immunoreactive cereal proteins (49). However, the quantification of gluten epitopes with this precise method can still be limited due to the high cereal protein polymorphism and an incomplete gluten database of oat immune responsive proteins (50).

The overall aim of our study is to demonstrate the variability of oat protein composition directly related to health-related and techno-functional properties. In this first report, we summarize our findings related to genetic factors in an international population of different oat cultivars that have been analyzed using a complex relatively fast and cost-effective protein separation methodology, suitable for characterizing large sample populations, and the resulting data have been evaluated, applying published proteomic information. While the data collected in this study on the overall protein composition, including the ratio of polymeric to monomeric oat proteins, can be directly related to functional properties, the results of the detailed analysis of avenin proteins can help breeders to select oat lines with suitable storage protein composition. The application of the same techniques, monitoring the effects of growing conditions on the protein composition of oat as well as the relationships between the protein composition and the techno-functional properties, is in progress and planned to be reported in subsequent publications.

## Materials and Methods

### Plant Material

In this study, 162 oat cultivars and breeding material were analyzed with different genetic backgrounds and places of origin, 37 from Australia, 2 from Belgium, 9 from Canada, 4 from Chile, 5 from China, 1 from England, 1 from Ecuador, 2 from Finland, 4 from Germany, 2 from Holland, 40 from Hungary, two different regions and breeding backgrounds (Szeged and Martonvásár), 2 from Japan, 2 from New Zealand, 2 from Peru, 2 from Poland, 7 from South Africa, 5 from Sweden, 34 from USA, and 1 from Uzbekistan. All of the names of the varieties are coded with the first three letters of the origin plus a running number to comply with proprietary issues and breeding licenses. For easier handling and interpretation of the large dataset, eight subpopulations (R1-R8) were created from all of the analyzed varieties, based on, more or less, the geographic origin of the samples that served as a basis of data evaluation (Supplementary Table 1). The oat samples were derived from small plot field growing. After harvest, samples were stored in a dry and cold warehouse. The dehulling was made with Satake grain testing mill TM-05 (Satake Engineering Co. Ltd., Japan), dedicated only to GF grains, and grinding of hulled grains was carried out with a Retsch MM 400 ball mill (Retsch GmbH, Germany) in a gluten-free laboratory environment, which was monitored with the R-Biopharm RIDASCREENRIDA®QUICK Gliadin test stripes (Art. No.: R7003).

### Protein Content

The protein content of oat flours was determined by the Dumas method (N × 5.95), an adaptation of the AOAC official method (51) using an automated protein analyzer (LECO FP-528, USA).

### Characterizing the Protein Composition of Cultivars by Size Exclusion-High Performance Liquid Chromatography (SE-HPLC)

Size exclusion-high performance liquid chromatography analyses have been carried out with three replicate injections from two replicate extracts. A simplified version of the procedure of Gupta et al. (52) was applied as a one-step extraction. Based on preliminary studies, it was found that more than 95% of the proteins of oats can be extracted by simply vortexing the samples, so in contrast with the observations in the case of wheat, there was no need for a second consecutive extraction step using sonication The size exclusion-high performance liquid chromatography (SE-HPLC) using the procedure of Batey et al. (53) was used as modified by Larroque and Békés (54) with a mixture of two stock buffer solutions: A (12 g of 0.2 M NaH_2_PO_4_ + 500 ml MQ H_2_O) and B (17 g of 0.2 M Na_2_HPHO_4_ + 500 ml of MQ H_2_O). The final SE buffer solution was prepared by mixing 90 ml of solution A + 110 ml of solution B + 600 ml MQ H_2_O + 4-g SDS.

Single grains from different samples were placed in 2 ml Eppendorf tubes with a 72-mm-diameter steel ball bearing placed on top of the grain. The tubes were lysed using a Qiagen®TissueLyser II (Qiagen GmbH, Germany) at 27 strokes/s frequency for 7 min. Flour from each tube (10 mg) was weighed in fresh 2 ml Eppendorf tubes, and 1 ml of an SE-HPLC extraction buffer was added to each tube. The tubes were then vortexed, using MO BIO Laboratories, Inc. Vortex-Genie®2 at setting 6 for 30 min. They were subsequently centrifuged for 15 min at 13,000 rpm, using Eppendorf Centrifuge 5424. The supernatant was then aspirated using a 1 ml syringe. The supernatant was then passed through a 0.45 μl filter into an HPLC vial. The vials were placed in an Agilent Technologies 1200 series HPLC instrument and were analyzed using the following parameters: a Mobile Phase of 50% acetonitrile (ACN), HPLC grade, with 0.1% trifluoroacetic acid (TFA) and 50% water HPLC grade, with 0.1% trifluoroacetic acid (TFA) was used. The SE column (Agilent AdvanceBio Sec 300A, 2.7 μl, 4.6 × 300 mm) was washed for 60 min with 100% water to 100% acetonitrile and stabilized for 1 h before commencing the analysis. The column was used at room temperature, at 120-bar pressure; the injection volume was 10 μl at a flow rate of 0.350 μl/min. The SE-HPLC separation resulted in 10 peaks (P1-P10), polymeric globulin proteins eluted first (P1-P5), avenins in P6 fraction, while the four latest eluted little peaks (P7-P10) (integrated together) contained the soluble non-avenin proteins.

### Reversed-Phase High-Performance Liquid Chromatography (RP-HPLC)

About 60 mg oat flour was extracted using 70% ethanol and vortexed in a horizontal vortex (Vortex-Genie® 2, MO BIO Laboratories, Inc., USA) at setting 6 for 30 min. Samples were centrifuged for 15 min at 13,000 rpm g using Eppendorf Centrifuge 5,424. The supernatant was aspirated with taking care of the pellet and passed through a 0.45 μl filter into an HPLC glass vial. The samples were prepared in triplicate and were centrifuged for 20 min at 15870 × g. The supernatant was filtered using a 0.45 μm filter. The protein extracts were separated using Agilent 1200 LC Systems (Agilent Technologies, USA) by the method of Larroque et al. (55). About 10 μl of extracts were injected into a C18 reversed-phase ZORBAX 300SB-C18 column (4.6 mm × 150 mm, 5 μm, 300 Å, Agilent Technologies, USA), maintained at 60°C column temperature and at 50-bar column pressure. The applied eluents were 67% ultrapure water (Buffer A1) and 33% acetonitrile (Buffer B1), each containing 0.1% TFA (HPLC grade, Sigma Aldrich). The separation was carried out using a linear gradient from 33 to 80% Buffer B1 over 65 min at a flow rate of 1 ml/min.

RP-HPLC analyses have been carried out with three replicate injections from two replicate extracts.

### R-Biopharm RIDASCREEN R5 ELISA Analyses

In order to detect gluten contamination from wheat, rye, or barley, oat samples were analyzed with the R-Biopharm RIDASCREEN® Gliadin assay (catalog number: R7001, R5 monoclonal antibody, sandwich format, LoD: 0.5 mg/kg gliadin or 1 mg/kg gluten, LoQ: 2.5 mg/kg gliadin or 5 mg/kg gluten). Extraction and the ELISA procedure were carried out in line with the kit instructions, adapted to local laboratory equipment. Briefly, 1 g of oat flour samples was weighed in 50 ml Falcon tubes. About 10 ml Cocktail solution (R-Biopharm, catalog number: R7016) was pipetted to each sample under a chemical hood. After vortexing, the samples were incubated at 50°C for 40 min in a shaking water bath (OLS Aqua Pro, Grant Instruments, United Kingdom). After cooling the samples to room temperature, 30 ml 80 V/V% ethanol was added to the samples, followed by 1 h of shaking on a table-top shaker (1,500 rpm, Vibrax VXR basic, IKA Werke, Germany). The samples then were centrifuged for 10 min at 2,500 × g at room temperature (LISA, AFI, France). Supernatants were diluted 1:12.5 with the sample diluent solution provided to the kit (the concentrate was pre-diluted prior to use according to the kit manual). About 150 μl of kit standards and samples were loaded to a transfer plate in duplicate. Finally, 100 μl of each sample and standard was transferred to the ELISA plate with a multichannel pipette. The plate was incubated for 30 min at room temperature and then was washed with the pre-diluted wash buffer provided for the assay in line with the kit instructions (ELx50 automatic plate washer, BioTek, USA). Then, 100 μl of the pre-diluted conjugate was added to all wells followed by 30 min of incubation at room temperature. After washing, 50 μl substrate and 50 μl chromogen were added to all wells, and the plate was incubated for 30 min at room temperature covered by aluminum foil. Finally, 100 μl of stop solution was added to all the wells, and absorbance values were obtained at 450 nm using a plate spectrophotometer (iMark, BioRad, USA). Data were analyzed with the Microplate Manager 6 software (BioRad, USA) using the cubic spline fit to create a standard curve. The results were the subject of further calculations to obtain the reporting unit of mg/kg gluten as per the kit instructions.

### Prediction of Avenin-Epitope Levels

The immunodominant T cell epitopes of oat DQ2.5-ave-1a (PYPEQEEPF), DQ2.5-ave-1b (PYPEQEQPF) (56, 57), DQ2.5-ave-1c (PYPEQEQPI) (48), and DQ2.5-ave-2 (PYPEQQPF) were predicted, and the epitope containing avenin levels in different oat varieties was calculated based on the study by Tanner et al. (58). Sollid et al. (47) determined the celiac disease–relevant, internationally agreed T cell epitopes recognized by CD4^+^T cells, namely, DQ2.5-ave-1a, DQ2.5-ave-1b, and DQ2.5-ave-1c. The study of Tanner even included the DQ2.5-ave-2 that contained only the minority of the investigated oat varieties, and the prediction was made based on it.

Briefly, Tanner et al. carried out RP-HPLC analysis from an Australian oat variety (cv. Wandering). The representative RP- HPLC chromatogram of the purified oat protein sample contained 18 well-defined RP peaks. RP-HPLC fractions were collected from the purified avenin sample and using MALDI-TOF-MS, and LC-MS/MS analysis of the chymotrypsin digested samples was carried out for protein identification. RP-HPLC analysis in this study has been carried out using the identical protocol in the same laboratory by the same operators as reported by Tanner et al. (58), resulting in matched elution profiles of avenin peaks with the published data and those derived from this study. The mass spectrometric information on the avenin peaks eluted at certain retention times the work of Tanner has been adopted to characterize the corresponding RP-HPLC peaks in our study. The individual and cumulative amounts of avenin proteins containing the four oat avenin T cell epitopes have been determined by selecting and summing the peak intensities based on the retention times of the peaks, expressed in [mg/100 g avenin] units using the average molecular mass of avenin proteins as 29 kDa (43) and with the molecular mass values of the four avenin epitopes, calculated from their amino acid composition and, finally, converted to [mg/100 g sample] units by multiplying the mg/100 g avenin values by the SE-HPLC-based avenin content and by the protein content of the samples.

Using proteomics data of Tanner in such a way is based on the assumption that their data, which are based on the detailed study on a single cultivar (cv. Wandering), is representative for oat cultivars in general. The approach to the prediction of epitopes from RP-HPLC data is strictly reliable when these data would be supported and confirmed by amino acid sequence data, demonstrating (at least in a representative number of cultivars), the actual presence and amounts of intact avenin epitope sequences in the distinguished HPLC peaks. With the lack of such data, the predicted epitope levels can be interpreted as the measure of the possible variation of epitope contents in the cultivars in the sample population rather than the exact epitope levels in the individual samples.

The cumulative amounts of the presumably immune reactive avenin proteins per variety were determined and expressed as a percentage of the sample mass by combining the peak data of RP- and SE-HPLC separation and protein content of the samples.

### Statistical Analyses

In the cases of both SE- and RP-HPLC analyses, mean values, standard deviation, and coefficient of variation have been calculated based on the six replicate data derived from the three replicate injections of two replicate extracts. The calculations have been carried out using MS Excel functions. Sample groups have been characterized by the variation of the above-mentioned mean values of different protein compositional data. To avoid any possible confusion, different notations are used for describing the variation among the replicate measurements of a given sample (mean, stdev, and cv) and the variation among the means of different measurements in a group of samples (mean, stdev, and cv).

In case of parameters derived more than one, standard deviations were calculated based on the Gaussian error propagation law (59) from the means and standard deviation values (**σ**) from the individual parameters: in case of the cumulative amount of epitopes, the geometrical mean of the four standard deviations were used while the following equation was used for the determination of the standard deviation of the avenin levels in mg/100 g samples unit:


$$\begin{array}{l} \sigma_{\text{amg}/100\text{ g sample}}=10^{-4}*mean_{{\text{protein}}^{*}}[{\left(\sigma_{\text{avenin}}\right)}^{\text{2}}*\\ {*(mean_{\text{cum}.\text{epitop}})\text{+}{\left(\sigma_{\text{cum}.\text{epitop}}\right)}^{\text{2}}*{\left(mean_{\text{avenin}}\right)}^{\text{2}}]}^{\text{0}.\text{5}} \end{array}$$

RP-HPLC profiles of the samples have been compared using pattern recognition techniques. The PATMATCH software (60) has been used for matching the chromatograms and identifying the corresponding peaks based on their elution time. Variation of retention times of peaks observed among replicate analyses and the minimum differences between the mean values of individual peaks have been determined and used to match the corresponding peaks from different samples. Similarity matrices using the presence and absence of peaks with the same elution time (S%) or with relative amounts of these individual peaks (S'%) have been constructed, also applying the PATMATCH software (60):


$$\begin{array}{l} S_{A,B}\%=100^{*}\left(\frac{2^{*}n_{A,B}}{n_{A}+n_{B}}\right)\\ {S^{\prime }}_{A,B}\%=100^{*}\left(\frac{2^{*}\sum_{n_{A,B}}^{i=1}e_{i}}{n_{A}+n_{B}}\right) \end{array}$$

where **n**_**A**_ and **n**_**B**_ are the number of peaks in samples A and B, **n**_**A, B**_ is the number of peaks with identical elution times in samples A and B, **e**_**i**_ is a weighting factor describing the relative intensity of peaks with identical elution time. Cluster analysis was carried out applying the similarity matrices with the Morpheus R package (https://software.broadinstitute.org/morpheus/).

ANOVA test and multiple comparisons of mean values based on the least significant difference (LSD) by Student *t*-test were carried out as implemented in the NCSS 2021 Statistical Software (2021), (NCSS, LLC. Kaysville, Utah, USA, ncss.com/software/ncss).

## Results

Protein composition of the oat flour samples has been characterized on two levels: distribution of the total protein content after size-based separation was determined with SE-HPLC, followed by the RP-HPLC-based determination of the qualitative and quantitative composition of the avenin fraction.

### SE-HPLC Analyses

More than 99% of the total amount of oat flour proteins has been extracted in the first step of the extraction procedure of Gupta et al. (52), without applying sonication. Comparison of samples has been carried out, therefore, using this simplified one-step procedure.

**Figure 1:**
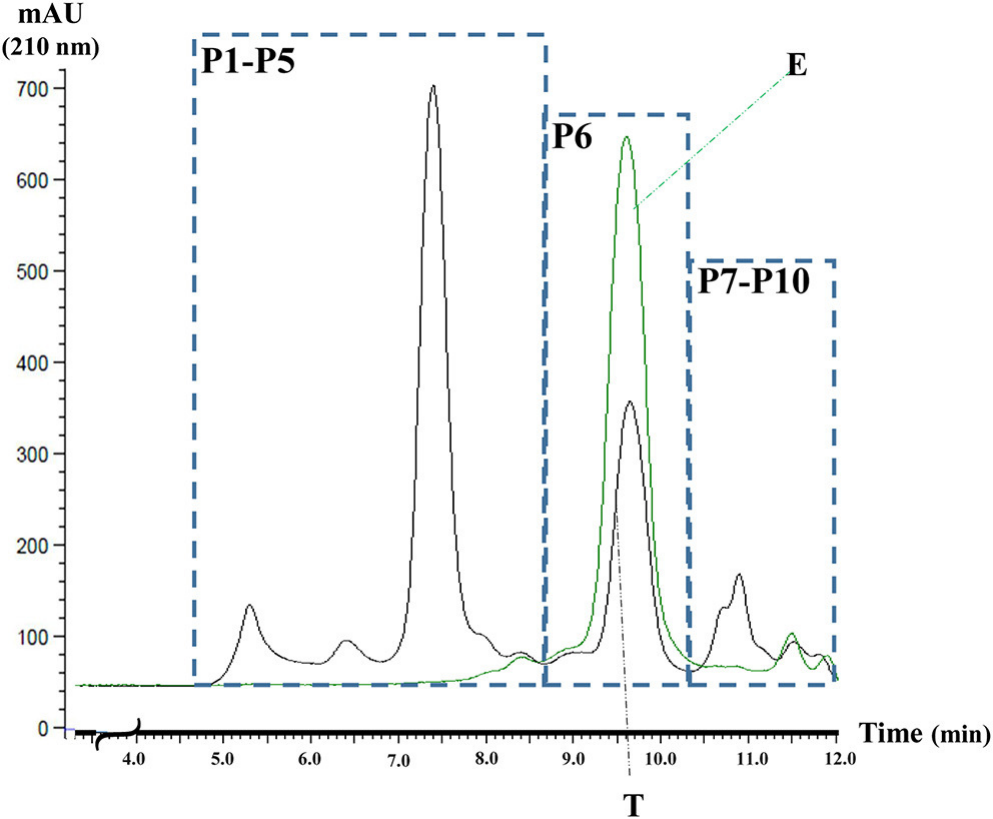
The typical SE-HPLC profile of the total oat protein extract (T) and 70% ethanol extract of oat flour (E). AU, absorbance units at 210 nm, P1-P5- polymer fraction, P6-avenins, and P7-P10-monomer globulins.

Three main protein groups have been detected based on the SE-HPLC separation (Figure 1). The polymeric protein fraction consisting of five well-defined peaks (P1–P5) with retention times of 5.2, 6.4, 7.4, 7.9, 8.3 min, respectively. The next main group is the avenin-type proteins, labeled P6 in Figure 1 (retention time: 9.6 min), while the third group, containing a rather complex mix of the monomer globulin proteins (P7–P10), eluted in the region of 10–12 min. The elution profile of the 70% ethanol extract is also shown in Figure 1, clearly indicating that the ethanol-soluble proteins are eluted as one single peak (P6), analyzing the total protein extract.

The reproducibility of the peak intensity measurements has been monitored by calculating the mean, stdev, and cv values for each peak from their six replicate analysis data (Supplementary Table 2). Based on the averages of cv values calculated from the data of the 6 replicates among the 162 samples, the overall errors for the polymeric, avenin, and non-avenin monomeric protein group measurements are 5.018, 6.016, and 7.145%, respectively.

The distribution of the proteins among the three main groups and inside of the polymeric fraction shows a well-defined trend all around the 162 samples. The polymeric fraction represents about three-quarters of the total protein content (Mean: 73.14%, min: 63.29%, max: 86.60%); the amount of the avenin fraction is varied between 8.86 and 27.72% (mean: 19.38%), while the amount of the monomeric globulin fraction is between 2.89 and 11.85% (mean: 7.29). In each sample, the relative amounts of the five subfractions of the polymeric proteins show a P1 < P2 > P3 >> P4 ⋙ P5 trend.

Comparing the relative distribution of the proteins in the different geographic regions (R1-R8), it was found (Table 1) that the total amount of polymeric proteins and its distribution among the five subfractions (with the exception of P2), the amount of monomeric globulin proteins, and the ratio of the polymeric to monomeric globulin proteins, show significant differences among the eight geographic groups.

**Table 1:** Statistical analysis on the variation of the size-based distribution of the total proteins of oats samples among the different geographic regions.

**SE-HPLC**	**Region**	**R1**	**R2**	**R3**	**R4**	**R5**	**R6**	**R7**	**R8**	**F**	***p***
**fraction**	***n***	**39**	**11**	**43**	**7**	**7**	**7**	**40**	**8**		
P1	mean	25.65	14.74	15.52	15.57	16.30	14.48	17.35	14.01	34.54	0.0000
		(b)	(a)	(a)	(a)	(a)	(a)	(a)	(a)		
	min	14.92	11.30	10.31	14.03	15.14	12.45	11.63	10.48		
	max	39.25	17.30	20.62	18.74	17.35	15.79	23.79	16.38		
	Sd	5.34	1.99	2.76	1.71	0.83	1.08	3.08	2.14		
P2	mean	34.67	35.92	36.65	35.25	35.36	35.67	35.05	35.97	0.43	0.8799
		(a)	(a)	(a)	(a)	(a)	(a)	(a)	(a)		
	min	13.37	31.14	30.70	32.80	32.73	32.85	7.37	32.06		
	max	47.80	39.32	42.16	39.52	39.26	37.12	49.52	39.33		
	Sd	6.01	2.29	2.19	2.50	2.06	1.55	8.92	3.10		
P3	mean	6.29	13.51	12.88	12.43	13.86	10.55	8.94	13.79	12.24	0.0000
		(a)	(b)	(b)	(b)	(b)	(ab)	(a)	(b)		
	min	3.56	11.35	6.43	10.40	12.13	9.43	3.19	10.31		
	max	12.91	16.26	17.39	13.95	15.28	12.19	34.22	18.87		
	Sd	2.32	1.79	2.48	1.39	1.09	1.00	6.81	2.44		
P4	mean	3.77	2.17	2.20	2.23	2.17	2.40	4.81	2.34	4.80	0.0001
		(ab)	(a)	(a)	(a)	(a)	(a)	(b)	(a)		
	min	0.26	1.93	1.61	2.04	1.96	2.22	2.23	1.94		
	max	8.31	2.55	3.00	2.46	2.33	2.58	9.56	2.95		
	Sd	1.33	0.20	0.30	0.18	0.14	0.16	0.46	0.37		
P5	mean	4.73	5.16	5.23	5.29	5.15	5.70	8.50	5.55	6.93	0.0000
		(a)	(a)	(a)	(a)	(a)	(a)	(b)	(a)		
	min	1.94	4.58	3.81	4.84	4.65	5.28	2.93	4.60		
	max	6.61	6.05	7.13	5.85	5.54	6.13	19.99	7.00		
	Sd	1.22	0.48	0.72	0.43	0.33	0.38	0.52	0.87		
	mean	75.10	71.50	72.47	70.77	72.84	68.79	74.65	71.66	4.15	0.0003
Polymers		(c)	(ab)	(b)	(ab)	(b)	(a)	(c)	(ab)		
	min	66.13	68.76	63.29	66.56	70.39	65.52	64.27	67.67		
(P1–P5)	max	84.02	74.35	80.23	73.32	76.19	71.08	86.60	76.84		
	Sd	4.36	1.63	3.52	2.72	1.92	2.01	5.25	3.44		
	mean	18.32	19.84	19.85	20.33	19.78	21.88	18.98	20.09	1.66	0.1240
Avenins		(a)	(a)	(a)	(a)	(a)	(a)	(a)	(a)		
	min	8.86	17.61	14.65	18.61	17.87	20.30	10.08	16.89		
(P6)	max	27.72	23.23	26.62	22.47	21.27	23.56	27.06	24.31		
	Sd	3.97	1.85	2.54	1.64	1.25	1.44	3.79	2.81		
	mean	6.56	8.66	7.67	8.90	7.39	9.32	6.37	8.25	7.21	0.0000
Monomers		(a)	(b)	(ab)	(b)	(ab)	(b)	(a)	(b)		
	min	4.08	6.69	4.28	7.66	5.94	8.60	2.89	5.47		
(P7–P10)	max	9.47	11.85	10.88	10.99	8.98	10.92	10.23	10.35		
	Sd	1.24	1.37	1.65	1.25	1.05	0.90	2.18	1.56		
Polymer	mean	11.89	8.44	10.00	8.10	10.06	7.45	13.47	9.05	6.70	0.0000
to		(bc)	(a)	(b)	(a)	(b)	(a)	(c)	(ab)		
monomer	min	7.35	5.89	6.27	6.05	7.84	6.00	6.41	6.63		
ratio	max	19.39	10.49	18.29	9.33	12.82	8.25	27.82	14.04		
	Sd	2.68	1.27	2.77	1.28	1.69	0.83	5.55	2.32		

*P values highlighted in red indicate significant differences among groups. Different letters indicate significantly different mean values based on Student t-test (p < 0.05)*.

Compared to the data in the rest of the geographic groups, the highest amount of polymeric proteins (means: 75.10 and 74.65%) and polymeric to monomeric protein ratio (means: 11.89 and 13.47%) were found in the R1 and R7, respectively. The cause of these values derived from significantly higher amounts of P1 fraction found in the R1 and R7 groups (means: 25.65 and 17.35%, respectively.), compensated only partly with the significantly lower values of P3 (6.29 and 8.94%, respectively) in these groups.

Beyond the apparently uniform avenin levels observed at the comparison of mean values in the different geographic groups, some extremely low (AUS05: 8.86%) and extremely high (AUS14: 27.72%) avenin contents were observed, for example, in the R1 sample group. These cultivars could have great potential to be applied to nutrition-related breeding programs.

### RP-HPLC Analysis

The RP-HPLC patterns and peak distributions showed great variation in the number and composition of different avenin polypeptides, indicating the extent of genetic and proteomic diversity in this large oat population (Supplementary Table 3). In the 162 oat samples, 76 distinct peaks have been matched by the PATMACH software in the 25.75 to 47.25 min elution time interval using a 0.10 min window to identify the corresponding peaks in the different chromatograms. It means that, if the differences in retention times of a particular peak in different samples were lower than 0.10 min, then the peaks have been evaluated as identical peaks. Using this procedure, the number of peaks in a given sample has been determined, indicating a large variation between 6 and 18 peaks (Mean: 10). This variation in the number of separated peaks can be explained by the variability of the resolution of RP-HPLC technique as the function of the amounts of proteins in a peak: the individual peaks in certain cases might contain more than one protein type (as it was shown in the work of Tanner et al. (58), characterizing individual RP-HPLC peaks by using the mass spectrometric methodology.

As it was observed in previous studies (for example, Tanner et al., 2019), most of the avenin polypeptides are eluted in two elution time intervals: 20 peaks have been found in the 25.75-32-min interval and 37 in the 38–47.25-min interval, representing the 45.58 and 48.42% of the total avenin content, respectively.

The number of appearances of a peak with a given retention time in different samples was found to be extremely variable. There are three peaks with the retention times of 25.75, 34.50, and 35.00 min found only in three cultivars, namely in US12, HUN25, and CAN06; 17 peaks have been identified, which appeared in less than 6 samples, while the peak with the retention of 42.39 min was found in 107 samples.

The level of large polymorphism of avenin polypeptides in the sample population investigated in this study is well demonstrated by the S% similarity matrix (not shown) and the cluster analysis diagram (Supplementary Figure 1). Based on the dendrogram, six clusters (A to F) can be identified characteristically containing or missing certain peaks indicated in Table 2 with bold or with italics, respectively. As the color scale of the diagram clearly indicates, similarities among samples in the cluster are significantly larger than those in any other clusters. The list of the clusters for the different samples is indicated in the last column of Supplementary Table 3.

**Table 2:** Characteristic peaks in the six similarity clusters of avenin protein RP-HPLC profiles and the origin-based distribution of oat samples among the clusters.

		**Characteristic peaks**							**Origin**	**R1**	**R2**	**R3**	**R4**	**R5**	**R6**	**R7**	**R8**
		**Retention time (min)**							**n**	**39**	**11**	**43**	**7**	**8**	**7**	**40**	**7**
**Cluster**	**A**	*27.80*	**30.64**	*43.33*	**43.67**				38	36	2						
	**B**	**27.00**	**28.78**	**29.09**	**29.32**	*30.12*	**43.10**	*43.33*	8							8	
	**C**	**29.09**	*29.32*	*30.64*	**40.50**	*43.67*			36		4	15	2	2	5	6	2
	**D**	**28.78**	*29.09*	**43.33**	*43.67*	*44.58*	**45.66**	**46.33**	26	1	2	10	2	4		3	4
	**E**	**28.78**	*29.09*	*31.09*	**40.50**	*43.10*	*43.67*		33	1	3	17	2	2	2	5	1
	**F**	*29.86*	**30.12**	*30.64*	**32.11**	**42.39**	**43.33**	*44.58*	21	1		1	1			18	

*Retention times in bold indicate peaks existing in a cluster, while retention times in italics indicate no peaks in a cluster. Blank fields indicate that, in that certain subpopulation, the characteristic RP profile defined is absent*.

Some interesting observations can be made, investigating the distribution of samples in the different clusters based on their origin (Table 2). While the samples in R2, R5, and R8 groups are scattered in different clusters, most of the samples in R6 group are together in Cluster C, the ones in R3 either C, D, or E, but not in A or B cluster; 18 from the 40 samples in R7 can be found in Cluster F, and 36 from the 39 samples in R1 are located in Cluster A.

Differences among the avenin composition of the samples are significantly enlarged if the amounts of the different peaks are used in similarity calculation (S'%) instead of the presence/absence-based comparison (S%). Expression levels of avenins with the same retention times in different samples have been found largely not uniform among the peaks.

The reproducibility of the peak intensity measurements has been monitored through the 1,530 peaks found in the whole sample population by calculating the mean, stdev, and cv values for each peak from their six replicate analysis data, resulting in a 7.18% for the average value for the cv values. The *r*^2^ value between elution times and cv values of peak intensities of peaks eluted at a given elution time was found to be 0.0036, while a strong negative correlation was found between the peak intensities, and their reproducibility (*r*^2^ = 0.7934): in the 10–15% peak intensity interval, the cv values are smaller than 6%, while in 6, 7 and 10, 11% in the 5–10 and 10–15% intensity intervals, respectively.

### Predicting the Amount of Celiac-Related Oat Epitope-Containing Components

Applying the data provided by Tanner et al. (58) for the composition of avenin fraction of the oat variety cv., the amounts of the celiac-related oat epitope-containing components of the 162 oat samples have been predicted based on their RP-HPLC analysis results.

Six dominant peaks were identified, containing conserved avenin types: peak 3 (R.T. = 28.133 min) in 43 samples, peak 6 (R.T. = 30.465 min) in 80 samples, peak 8 (R.T. = 31.152 min) in 60 samples, peak 15 (R.T. = 44.158 min) in 36 samples, peak 16.2 (R.T. = 44.408 min) in 48 samples, and peak 16.3 (R.T. = 44.914 min) in 79 samples. Peak 3 contained the gliadin-like avenin (L0L6J0), peak 6 contained, also, a gliadin-like avenin (L0L6K1), peak 8 contained an Asat-Prolamin10 protein and a 23539 Da avenin (Q09072), peak 15 contained an avenin-F protein, with an alternative name celiac immunoreactive protein 2 or gamma-avenin-3 (Q09097) and an Asat-Prolamin71 protein, peak 16 contained an avenin (I4EP54), a gliadin-like avenin (L0L6J0), and an Asat-Prolamin15 protein. In the case of peaks 3, 6, and 8, the predominant avenin epitope is the DQ2.5-ave-1a (PYPEQEEPF), in peak 15, the DQ2.5-ave-1b (PYPEQEQPF) and DQ2.5-ave-1c (PYPEQEQPI), while, in peak 16, all the above mentioned three avenin epitopes occurred.

The individual and cumulated amounts of avenin epitopes have been determined by selecting and summing the RP-HPLC data according to their retention time, and then converting the resulting values to epitope contents based on their molecular mass. Finally, these values in [mg/100g total avenin] have been converted to [mg/100 g sample] units. Mean values, standard deviations, and cv values were calculated from the six replicate RP-measurements together with the protein content of the samples and six replicate SE-HPLC data for avenin content in case of the conversion to [mg/100 g sample] unit – (Supplementary Table 4).

Satisfactory reproducibility has been observed for the individual and cumulated epitope levels (average cv values calculated for the 162 samples for the DQ2.5-ave1a, DQ2.5-ave11b, and DQ2.5-ave1c epitopes and for their cumulated value: 0.096, 0.067, 0.082. and 0.063, respectively). The cv values for the avenin levels expressed in [mg/100 g sample] units varied between 0.003 and 0.129 with an average of 0.062.

### R5 ELISA

For the pure oat line development study, a small population consisting of 32 Australian and 35 Hungarian samples (Supplementary Table 5) was selected from the basic population for ELISA testing. Samples were selected to cover a wide range of crude protein content using samples with sufficient available amounts. The presence of potential gluten contamination from other cereals was tested with the R5 ELISA method of R-Biopharm. Based on the results of this test, 19 Australian and 24 Hungarian samples of the investigated oat varieties were uncontaminated, thus, deemed appropriate for the requirements of pure oat cultivation in terms of purity. Our results confirm that gluten contamination of oats is a serious problem and must be carefully addressed when providing seeds for growing pure oats.

## Discussion

The aim of our work was to carry out a high-throughput analytical screening completed with immune analytic measurements to develop a reliable prediction method for estimating the amount of avenin proteins and those that contain celiac-related epitopes. This special prediction method utilizes the combined application of SE- and RP-HPLC separation of the total protein content of the oat flour samples and differentiates the absolute levels of the four main avenin epitopes of the samples, and also provides the celiac-related epitope, containing avenin content in the oat flour (g/100 g).

Most of the oat-related research in the last 10 years concentrated on avenins, debating on their harmfulness in relation to celiac disease. Meanwhile, oats started to be recognized as a healthy and nutritious cereal, containing a high concentration of soluble fiber (β-glucan) and being dense in nutrients. It has physiological benefits like reducing hyperglycemia, hyperinsulinemia, and hypercholesterolemia, and several other benefits are discussed in several reviews like the one by Ibrahim et al. (61).

Interestingly, no application of SE-HPLC on characterizing oat proteins is reported in the critical work of Sunilkumar and Tareke (62), which reviewed the analytical methods for measurement of oat proteins by covering 2,000 works published between 1970 and 2015.

However, the application of size-related analytical techniques like SE-HPLC has a large potential to be used in selecting oat lines for industrial ingredient use (61).

In the scientific literature, there are many useful high-throughput studies on the methods developed to estimate the immunoreactivity of oat avenins and the availability of safe oat varieties for patients with celiac. A combined method using RP-HPLC and electrophoresis of oat avenins has been reported earlier (63), and the utility of the RP-HPLC for the identification of oat varieties has been demonstrated (64). It has also been suggested that RP-HPLC of alcohol-soluble storage protein fractions would be useful for selecting oat varieties with reduced immunogenicity for patients with CD (42). Giménez et al. (65) differentiated 120 oat cultivars from five geographical origins based on RP-HPLC peak profiles of avenins, combined with G12 competitive ELISA. The researchers confirmed that the RP-HPLC technique is useful to establish groups of varieties, differing in degree of storage proteins with low immunoreactivity for patients with CD, but not sufficient to uniquely identify the different varieties of the set (65). Schalk et al. (66) presented well-defined gluten protein fractions and types of wheat, rye, barley, and oat flours using mixtures of four cultivars each to account for the genetic variability between different cultivars, including the most relevant cultivars in Germany 2012.

Souza and co-workers revealed that avenin patterns of the examined oat cultivars are not distributed equally based on the place of origin (67). Previous papers reported the connection between oat prolamins and disease resistance genes. Gimenez et al. (65) pointed out that, according to this correlated variation, environmental and breeding factors caused non-random avenin profile variability. The study aimed to evaluate how variable avenin protein patterns of different oat cultivars are linked with low avenin content. Colgrave et al. (68) developed a high-throughput and sensitive approach to identify the possible source of gluten-like proteins in the view of contamination of GF grain. It reveals that the examined commercial oat flour samples were, in fact, contaminated by trace amounts of wheat.

Based on the results of our study, the high variability of avenin fraction composition and biodiversity of cultivated oat varieties are in agreement with the results of several research groups who are experts of this field.

The key avenin peptides that stimulate the pathogenic gluten-specific T cells in patients with CD *in vivo* have been defined (48, 69). These peptides contain the immunodominant T cell epitopes DQ2.5-ave-1a (PYPEQEEPF), DQ2.5-ave-1b (PYPEQEQPF), DQ2.5-ave-1c (PYPEQEQPI), and DQ2.5-ave-2 (PYPEQQPF) with close sequence homology to barley T cell epitopes immunoreactive in CD such as DQ2.5-hor-3a (PIPEQPQPY) (69). Londono et al. (70) investigated 13 Avena species, and no perfect gluten epitopes were found in avenins; besides this, none of the R5 and G12 antibodies recognition sites were found. The ELISA assay is a widely used method that gives quantified information about the contamination level and traces the possible source of gluten-like proteins in cereal crops. ELISA R5 shows no cross-reactivity to oats and can, therefore, be used to assess wheat, rye, or barley contamination in oats. The study of Comino and co-workers allowed the classification of oat varieties into three groups based on their degree of affinity for the G12 antibody: a highly reactive group is not safe for patients with celiacs; the moderate recognition group is not recommended, and one with no reactivity is a potential celiac safe group (46, 71). However, oat avenin extracts usually have a low G12 antibody response, the G12 reactivity well correlates with the results of T cell proliferation and interferon γ release. A direct correlation of the reactivity with G12 and the immunogenicity of the different prolamins were observed (72). In contrast, a comprehensive study by Londono and co-workers proved (70) that the signals of R5 and G12 should not be interpreted as differences in immunogenicity of oat varieties because of the lack of antibody recognition sites in avenins.

However, some preclinical studies working with cell cultures revealed differences in the immunogenicity of the different oat genotypes (46, 72); the results of the clinical investigations and data with organ culture system did not correlate, and refuted them (73, 74). Based on their results, oats do not display *in vitro* activities related to CD pathogenesis, and the T-cell reactivity could be below the threshold for clinical relevance, and it affects only a minority of patients. Besides this, researchers elaborated on the real CD-toxicity of the oat CD-immunogenic epitopes (48) and concluded that these have high protease sensitivity (22) and a relatively low HLA-binding capacity (48). Another research group has also demonstrated the sensitivity of avenins to proteolytic enzymes; DQ2.5-ave-1a and DQ2.5-ave-1c were completely digested by pepsin, trypsin, and chymotrypsin. The DQ2.5-ave-1b was proteolyzed by brush border enzymes (mostly by the prolylendopeptidase) (74). The susceptibility of oat avenins to proteolysis corresponds to their low-proline content (an average of 6% in avenins) (74). Both factors, together, significantly reduce the immunoreactivity of avenins and thus of oat-based foods. These findings were confirmed by the study of Hardy and co-workers in a large-scale oat challenge proved that the ingestion of oat is safe for patients with celiac without intestinal damage and serological relapse.

Because pure oat consumption carries a low risk for patients, the researchers declare that the strict control of production systems of pure oat is of utmost importance, and the regular follow-up of the patients with CD is recommended. Based on the R5 R-Biopharm RIDASCREEN® Gliadin assay of the selected subpopulation showed that 35% of the samples were contaminated. This highlights the necessity of improving the pure oat line and developing very sensitive and specific analytical methods for the sake of food safety.

All observations described above were derived from a reasonably large study where the carefully executed experiments were carried out with 2 × 3 replicates. The resulting data have been thoroughly analyzed statistically, taking into consideration the non-trivial characteristics of cumulative and complex parameters, where the actual results were derived from several independent measurements with experimental errors. The reproducibility of the two chromatographic separations, as well as the final cumulative results, seems to be satisfactory with the relative errors being under 12%.

These positive experimental characteristics, however, do not avoid two principal limitations of the prediction method introduced here:

- The reliability of the predicted information derived from this prediction process strongly depends on the validity of the assumption that the proteomic data (derived from the analysis of one single cultivar) are representative of oat cultivars in general. The predicted epitope levels should be validated by detailed proteomic analysis to avoid this limitation. With the lack of such validation, the predicted epitope levels can be interpreted as the measure of the possible variation of epitope contents in the cultivars in the sample population rather than the exact epitope levels in the individual samples.
- Because of the limited resolution of the RP-HPLC separation of avenin proteins, some oat polypeptides co-elute, producing false-positive results. Therefore, the predicted epitope levels have to be interpreted as upper limits.

In this study, large qualitative and quantitative differences have been observed in the avenin composition of the samples investigated: both the individual and cumulative amounts of the four oat avenin epitopes show large variation.

Analyzing the data, the most important observation is that, while certain cultivars do not contain all the four different epitopes, there is no variety among the 106 samples not containing any DQ2.5-ave epitopes.

Data shown in Table 3 were calculated from the mean values of replicate measurements (Supplementary Table 4); the average amount of the DQ2.5-ave-1a epitope in the samples is more than double compared with those of DQ2.5-ave-1b or DQ2.5-ave-1c epitopes (1501.28, 676.72, and 585.20 mg/sample), respectively. The number of cultivars where the presence of the individual epitopes has been demonstrated (Table 3) shows the same sequence: 104, 100, 93, and 3. The amount of DQ2.5-ave2 epitope in the three samples (US14, US31, and HUN13) where this epitope is present is marginal (34.75, 9.20, and 11.70 (mg/100 g sample)], respectively. Huge variation in the levels of the individual epitopes has been found, with larger than 0.5 cv values for each epitope class. The cumulative amount of epitope content in the samples varied between 2.20 and 270 mg in the 100 g sample with a strongly asymmetric distribution (Supplementary Figure 2), with the maximum number of 46 cultivars (28.40%), containing 26–50 mg/100 g epitopes. Two cultivars have been found with epitope levels of less than 5 mg/100 g (HUN31 and AUS04); these rarely found low levels could be utilized in breeding for healthy oat varieties.

**Table 3:** Variation of the amounts of celiac-related avenin epitopes among 106 oat samples.

	**DQ2.5- ave-1a**	**DQ2.5- ave-1b**	**DQ2.5- ave-1a**	**DQ2.5- ave2**	**Cumulative amount of celiac related avenin epitopes**	
	**mg/100 g avenin**					**mg/100 g sample**
Mean	1501.28	676.72	585.2	18.55	2763.2	84.92
min	0	0	0	0	103.84	2.20
max	3753.64	1651.76	1651.76	39.16	6900.52	270.60
StDev	859.76	419.76	432.52	14.09	1427.8	55.44
C.V.	0.57	0.62	0.74	0.76	0.52	0.65

As the results of the large variation of epitope levels in the whole sample population, significant differences among the origin-based subgroups can be observed (Table 4) for the amounts of DQ2.5-ave-1a and DQ2.5-ave-1c, but not for DQ2.5-ave-1b. The highest F value (5.6672) was found for the cumulative epitope levels data expressed as [mg/100 g sample] what can be explained by the fact that these values do not only derive from the variation in avenin composition, but they are varied by the total amount of avenin proteins as well as the protein content of the samples. The comparison of mean values, in this case, shows significantly lower levels in the Australian samples (47.52 mg/100 g sample) compared with the South African and South American samples (117.92 and 123.61 mg/100 g samples), respectively.

**Table 4:** ANOVA comparison on the predicted celiac-related avenin epitope contents of samples in the eight regions of origin.

		**mg/100 g avenin**								**mg/100 g sample**	
**Group**	**n**	**DQ2.5-ave-1a**		**DQ2.5-ave-1b**		**DQ2.5-ave-1c**		**DQ2.5-ave-1a** **+** **DQ2.5-ave-1b** **+** **DQ2.5-ave-1c** **+** **DQ2.5-ave2**			
R1	39	1441.00	(ab)	513.48	(a)	349.36	(a)	2304.28	(a)	47.52	(a)
R2	11	1744.60	(b)	697.84	(a)	552.64	(b)	2995.08	(ab)	96.36	(ab)
R3	43	1769.24	(b)	755.04	(a)	714.56	(bc)	3239.72	(b)	106.04	(ab)
R4	7	1930.72	(b)	841.72	(a)	830.72	(c)	3603.16	(b)	123.64	(b)
R5	7	1216.16	(a)	592.68	(a)	557.04	(b)	2365.88	(a)	82.28	(ab)
R6	7	1522.84	(ab)	722.04	(a)	658.24	(b)	2903.56	(ab)	102.64	(ab)
R7	40	1079.76	(a)	696.96	(a)	614.68	(b)	2392.28	(a)	77.88	(ab)
R8	8	1981.32	(b)	807.40	(a)	680.24	(b)	3468.96	(b)	117.92	(b)
	**F-Ratio**	3.0997		1.4139		2.8668		2.5627		5.6672	
	***p***	0.0044		0.2034		0.0077		0.0159		0.0001	

*Different letters indicate significantly different mean values based on Student t-test (p < 0.05). P values highlighted in red indicate significant differences among groups*.

**Figure 2:**
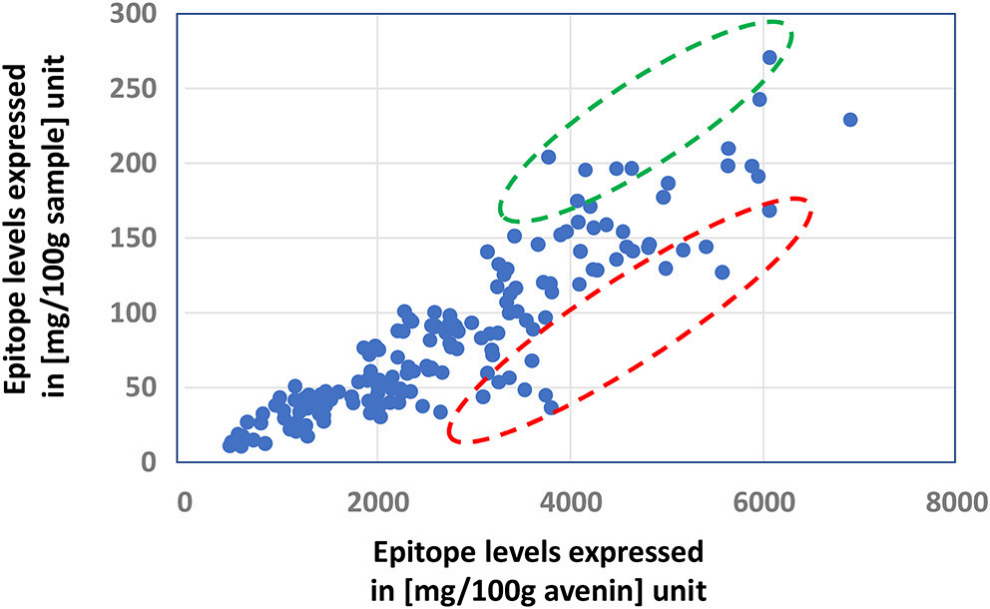
Demonstrating the importance of expression levels of avenin and non-avenin proteins in the ranking of relative celiac epitope amounts of oat samples by the comparison of ranking samples based on the amount of celiac-related epitopes expressed in (mg/100 g avenin) and (mg/100 g sample) units. Relative celiac-related epitope levels in samples in the red circle are largely underestimated by the simple comparisons of the epitope levels in the samples, not taking into account the total protein content and its avenin content. Circled data with red and green indicate under- and overestimated epitope levels using [mg/100 g avenin] units, respectively, not considering the contribution of protein content and avenin content of the sample.

The celiac-related epitope content of an oat sample is determined by its avenin composition, but the relative expression levels of both avenin- and non-avenin-type polypeptides can overwrite the ranking of the overall epitope levels in the samples, as it is illustrated in Figure 2: In the samples in the circled interval of the figure, the epitope levels expressed in mg/100 g avenin protein unit are misleading, underestimating the amount of epitopes taken by the consumed oat.

As it is well established for all cereal crops, including oats, both the protein content and protein composition are highly affected by the growing conditions, including both environmental and agrotechnical factors. Based on an unpublished large project carried out in our laboratory, investigating the alteration of the protein composition of 180 oat cultivars under rainfed and irrigated conditions, protein content of the samples of the same cultivar can be altered by 15 relative percentages while the ratio of polymeric and avenin proteins can vary by 38 relative percent caused by the water availability.

The observation illustrated in Figure 2 underlines the need for quantitative characterization of the overall protein composition rather than simply concentrating on the avenin composition, estimating the celiac-related epitope content of oat samples.

## Conclusion

Utilization of oats lines for human consumption requires the use of a reliable methodology of monitoring the presence and quantity of epitope containing components in the samples, and a better understanding of chemical composition and technological properties is needed. Both of these aspects require the active use of quantitative protein analytical techniques for the characterization of the whole spectra of oats proteins, albumins, globulins, prolamins, and glutelins. The application of detailed protein composition data has huge potential both in evaluating oats breeding lines in the pre-breeding selection phase and in monitoring oats-containing products in the food industry.

The combination of SE- and RP-HPLC methodology with active use of available proteomic data seems to be a satisfactory tool for these types of applications. Relating SE-HPLC-related quantitative protein analytical data to functional properties of oat samples like water and oil-binding capacity, emulsifying and foaming properties and even rheological properties of oats-containing doughs are in progress to utilize the data collected in this study.

Despite these valid and serious above mentioned limitations of the prediction method developed in this work, our view is that, with the lack of any other (better) relatively high throughput and cheap method, what is applicable to large sample populations the method is suitable to be used as a preselection screening tool in oat breeding in its present form already. Ongoing attempts to carry out further individual RP peak proteomic validation studies on different oat varieties, hopefully, will make our prediction method much more accurate in the future.

## Data Availability Statement

The original contributions generated for the study are included in the article/Supplementary Material, further inquiries can be directed to the corresponding author/s.

## Author Contributions

FB, ZBu, GG, and ST designed the study. KÁ, KS, BV, GV, and OV provided samples and sample preparation. FB, GG, CF, ZBu, DR, ZBi, ES, BL, ZS, EM, SP, and ST performed experiments and analyzed data. GG, ZBu, DR, KÁ, ZBi, and FB wrote the manuscript. All authors contributed to the article and approved the submitted version.

## Funding

The authors declare that this study received funding from the “GalgaGabona” Project (2017-1.3.1-VKE-2017-00004, Hungarian National Research, Development and Innovation Found), and from the BME-Biotechnology TKP grant of EMMI (BME TKP-BIO). GG was supported by the János Bolyai Research Scholarship of the Hungarian Academy of Sciences by the ÚNKP-18-4-BME-393, ÚNKP-19-4-BME-417, and ÚNKP-20-5-BME-292 National Excellence Program of the Ministry for Innovation and Technology from the source of the National Research, Development, and Innovation Fund. The funders were not involved in the study design, collection, analysis, and interpretation of data, the writing of this article or the decision to submit it for publication.

## Conflict of Interest

KÁ, BL, and SP were employed by company Cereal Research Non-Profit Ltd. KS was employed by company First Pest Mill and Bakery Ltd. FB was employed by company FBFD PTY Ltd. The remaining authors declare that the research was conducted in the absence of any commercial or financial relationships that could be construed as a potential conflict of interest.

## Publisher's Note

All claims expressed in this article are solely those of the authors and do not necessarily represent those of their affiliated organizations, or those of the publisher, the editors and the reviewers. Any product that may be evaluated in this article, or claim that may be made by its manufacturer, is not guaranteed or endorsed by the publisher.
