# Supplementary material for: Investigation of Protein and Epitope Characteristics of Oats and Its Implications for Celiac Disease
Source: Front Nutr. 2021 Sep 29;8:702352. doi: 10.3389/fnut.2021.702352 (PMC8511309; doi:10.3389/fnut.2021.702352)
Supplement: Supplementary file 6 [file Data_Sheet_1.PDF]

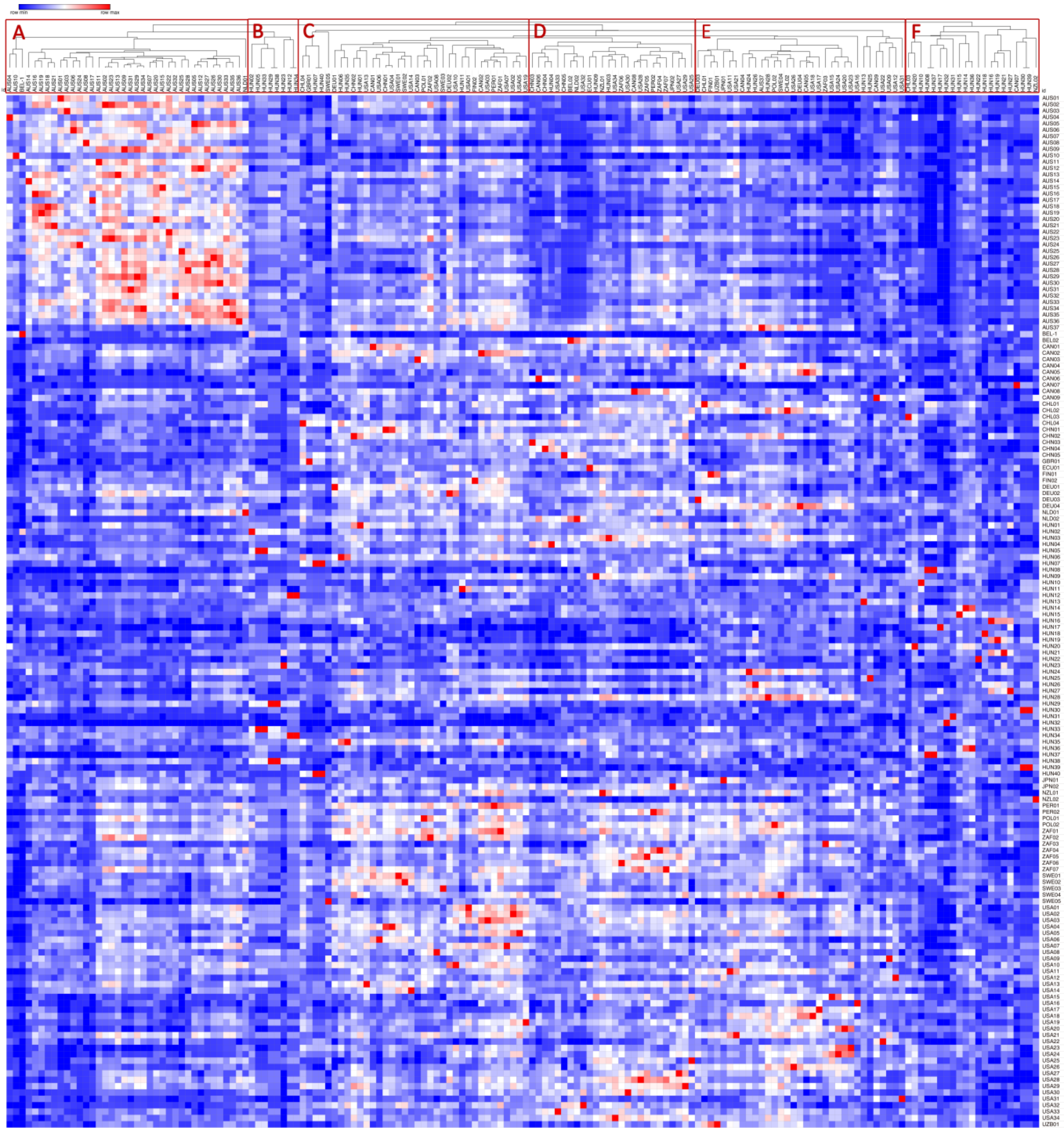

Supplementary Figure 1. Six clusters were defined by cluster analysis (A-F) based on the presence/absence of peaks in the RP-HPLC profiles of avenin proteins.

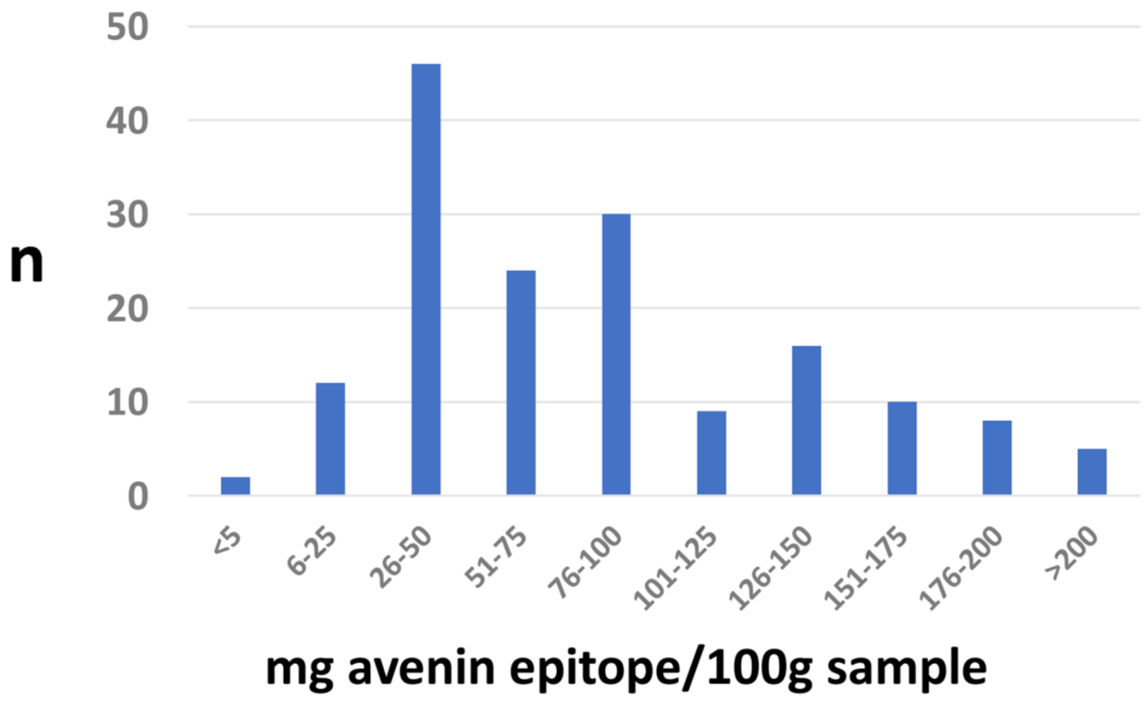

Supplementary Figure 2. Distribution of avenin epitope levels [mg/100g sample] among the sample population.
